# Nutrient availability in tropical caves influences the dynamics of microbial biomass

**DOI:** 10.1002/mbo3.1044

**Published:** 2020-05-11

**Authors:** Caio César Pires de Paula, Maria Elina Bichuette, Mirna Helena Regali Seleghim

**Affiliations:** ^1^ Post-Graduate Program in Ecology and Natural Resources Federal University of São Carlos São Carlos Brazil; ^2^ Department of Aquatic Microbial Ecology Institute of Hydrobiology Biology Centre CAS České Budějovice Czech Republic

**Keywords:** Brazil, decomposition, microbial parameters, organic matter, subterranean environment

## Abstract

Few studies have evaluated the trophic level in tropical caves, and none related the microbial biomass dynamics in the immobilization of carbon and nitrogen. Here, four tropical caves of Terra Ronca State Park, Brazil, were studied: Angélica, São Bernardo, Terra Ronca I, and Terra Ronca II caves. Physical, chemical, and microbiological parameters (microbial biomass and respiration) were estimated in the dry and wet seasons. São Bernardo, Terra Ronca I, and Terra Ronca II caves presented higher nitrogen and microbial biomass nitrogen (MBN) values in the wet season than in the dry season. On the other hand, the Angélica cave showed larger amounts of nitrogen and lower MBN values in the dry season. These results indicate that caves can be adjusted in two ecological theories known as “stoichiometric decomposition” and “microbial nitrogen mining”—to the effects of nutrient availability on organic matter decomposition. The caves studied showed different environmental dynamics in relation to organic matter decomposition, which allows them to be considered unique and possess specific characteristics. Microbial biomass dynamics can be an important parameter to evaluate the availability of nutrients and ecological dynamics of the trophic network in subterranean environments.

## Graphical abstract

Microbial biomass shows different decomposition patterns among tropical caves, which could be interpreted by two theories: stoichiometric decomposition and microbial nitrogen mining. Our results indicate that nutrient flow and ecological dynamics, such as microbial parameters related to decomposition processes, cannot be generalized to all subterranean environments.

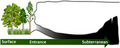

## 1 INTRODUCTION

Limestone caves are habitats in karst landscapes where surface water sinks into soluble rock in the subsurface and flows in a network of subterranean stream passages (Ford & Willians, 2013). Although hydrological flow regimes, watershed geometry, aqueous geochemistry, and bedrock geology differ between karst areas (Bonacci, Pipan, & Culver, 2008; Simon, Pipan, & Culver, 2007), many caves have similar characteristics and stable environmental conditions, such as temperature and humidity (Griebler et al., 2010; Hahn & Fuchs, 2009).

Many researchers consider temperate caves to be extreme oligotrophic environments (<5 mgC/L) (Engel, 2007). The partial or total absence of light in areas farther from the entrance and the limitation of resources contribute to the uniqueness of these places, resulting in microbial characteristics, such as the absence of phototrophic organisms. However, little is known about the flow of nutrients in subterranean systems (Gilbert, 1986; Simon, Benfield, & Macko, 2003), thus restricting the discussion about the limitation of resources in this ecosystem. Therefore, recent research in tropical caves shows that these subterranean environments may not be limited by the energy input (Paula, Montoya, Rodrigues, Bichuette, & Seleghim, 2016) since caves can receive a large input of organic matter through dripping water, floods of subterranean rivers and animals, and their excreta entering the caves (Jurado et al., 2010).

Microorganisms are important components of all subterranean ecosystems (Chapelle, 2000; Griebler & Lueders, 2009). In these environments, the microorganisms are key agents in nutrient flux dynamics (Simon et al., 2007) and can also regulate chemical reactions that cause mineral dissolution and precipitation (Engel & Randall, 2011; Lian, Yuan, & Liu, 2011). Some studies in aquatic and marine caves showed high microbial activity in the early stages of organic matter decomposition that correlated with increased microbial respiration (Fichez, 1991; Graening & Brown, 2003). Other studies highlighted the importance of the microbial community as key organisms at the base of the subterranean food web and in decomposition processes (Graening & Brown, 2003; Paula et al., 2016). Microbial parameters, such as biomass and microbial respiration, can be used as sensitive indicators to monitor changes in subterranean environments (Andrews, Karlen, & Cambardella, 2004; Matsuoka, Mendes, & Loureiro, 2003).

Microbial biomass is a living component that generally comprises 1%–5% of the total soil organic matter and plays a critical role in soil fertility because of its relatively fast turnover rate (Jenkinson & Ladd, 1981). The physicochemical characteristics of soil have a great impact on its microbial biomass and can be used to measure environmental quality (Parr & Papendick, 1997), but it may take years for these parameters to result in significant changes in the environment. Also, microbiological and biochemical changes are very sensitive to small changes in environmental conditions and thereby provide more accurate and immediate information on environmental quality because the microbial activity has a direct influence on ecosystem stability (Schloter, Nannipieri, Sørensen, & Elsas, 2018; Spohn, 2015).

Microbial respiration can be considered another important attribute in ecosystems, such as subterranean ones. Large amounts of organic carbon (C) are transformed, stored, and respired by microorganisms. Hence, new insights into the factors controlling the respiration rate per unit of microbial biomass are crucial for understanding the terrestrial C cycle (Zhou et al., 2014).

The studied area of this work (Terra Ronca State Park) has a complex system of superficial and subterranean drainage, with great potential for the transport of organic matter, causing debris accumulation in some cavities. Due to this, their cavities are highly rich in subterranean terrestrial and aquatic macrofauna taxa (Bichuette & Trajano, 2003; Pinto‐da‐Rocha, 1995; Rheims & Pelegatti‐Franco, 2003; Simões, Ferreira, & Bichuette, 2013; Trajano, Majer, Santos, & Basile, 2003). However, there are no studies on the dynamics of microbial biomass in this system and no other tropical cave. Thus, the objective of the present work was to study the dynamics of microbial parameters (biomass and respiration) and its relationship with nutrient availability (carbon and nitrogen) in four tropical caves of Terra Ronca State Park.

## 2 MATERIAL AND METHODS

### 2.1 Study site

Terra Ronca State Park (PETeR) (46°100′–46°300′S; 13°300′–13°500′W), located in São Domingos city (Goiás State, central Brazil), has a large subterranean system formed by rivers arriving from the Serra Geral Plateau, a morphologic feature originating in the sandstones of the Urucuia Formation (Cretaceous age). Large caves have developed along the trajectories of these rivers passing through Neoproterozoic carbonate formations from the Bambuí group (Guyot, Auler, Oga, Obstancias, & Appay, 1996). PETeR is a karst area crossed by parallel streams running westwards to join the Paraná River, a tributary of the Upper Tocantins River, in Amazonas Basin. The major streams and some of their tributaries cross the surface and enter a cave through a sinkhole, pass hundreds to thousands of meters through subterranean conduits, and surface through a resurgence. These are typical headwater streams, with rocky bottoms with gravel, pebbles, boulders, and transparent and well‐oxygenated waters.

The study area is inserted in the Cerrado phytogeographical domain (South America savannah‐like). The climate of the study area is tropical semi‐humid with a mean annual precipitation of approximately 1,270 mm/yr registered at the meteorological station in São Domingos‐GO (Moquet et al., 2016) close to the PETeR. The wet season extends from November to April, and rainfall essentially ceases between May and October (dry season). Floods may render some caves partially or entirely inaccessible in the wet season (November to April). Due to the high carrying capacity of these streams and the fact that the caves in the area represent stream sinkholes, substantial amounts of organic matter from vegetal debris and associated fauna accumulate inside these caves.

**Figure A1:**
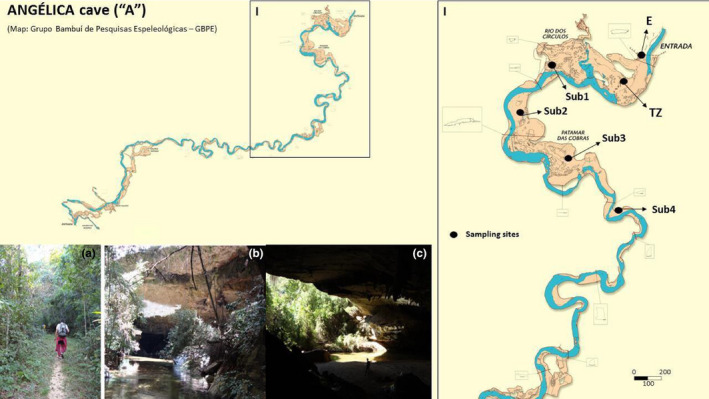
Angélica cave highlighting (frame I) the sites sampled: surface sites, entrance cave (E and TZ) and subterranean (Sub) environments. The images present the superficial environment (a), external (b) and internal (c) view of the entrance of the cave

**Figure A2:**
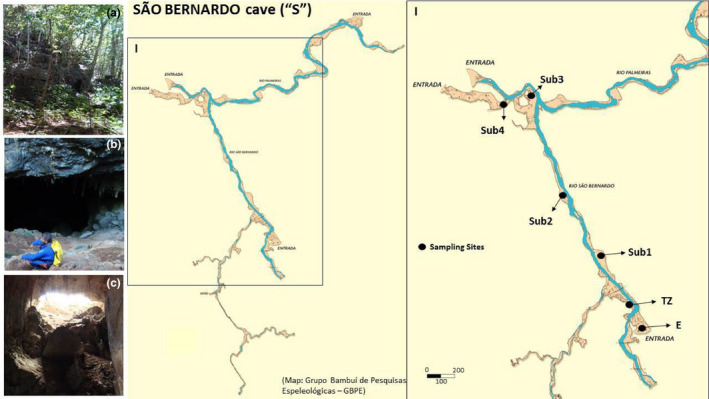
São Bernardo cave highlighting (frame I) the sites sampled: surface sites, entrance cave (E and TZ) and subterranean (Sub) environments. The images present the superficial environment (a), external (b) and internal (c) view of the entrance of the cave

**Figure A3:**
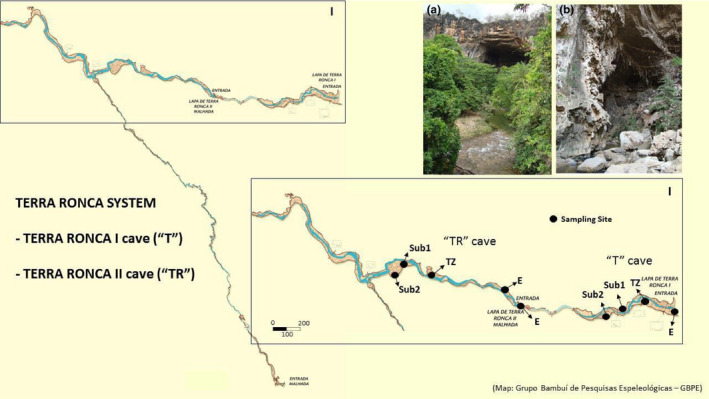
Terra Ronca System highlighting Terra Ronca I and Terra Ronca II caves (frame I), and the sites sampled: surface sites, entrance cave (E and TZ) and subterranean (Sub) environments. The images present the entrance of “T” cave (a) and the entrance of “TR” cave (b)

Four caves were studied: Angélica Cave (“A” cave), São Bernardo Cave (“S” cave), Terra Ronca I Cave (“T” cave), and Terra Ronca II Cave (“TR” cave). Angélica Cave (13°31’29” S and 46°23’07” W) is crossed by the Angélica River and is one of the largest caves of Brazil, with an extension of approximately 14 km and an entrance approximately 10.0 m height (Appendix Figure A1). It is part of a huge cave system with subterranean drainage named Angélica‐Bezerra (Bichuette, Simões, Schimonsky, & Gallão, 2015). São Bernardo cave (13.81°S and 46.35°W) is crossed by the São Bernardo and Palmeiras Rivers (São Bernardo – Palmeiras System) (Appendix Figure A2). São Bernardo cave entrance is a doline located at an altitude of 631 m.a.s.l. This cave has a great aquatic and terrestrial macrofauna diversity with endemic and troglomorphic species, and it is considered a hot spot of biodiversity (Bichuette et al., 2015; Trajano et al., 2003). Terra Ronca I and Terra Ronca II caves are part of the Terra Ronca‐Malhada system (Appendix Figure A3). Thousands of years ago, a landslide caused its division into two parts. Currently, a canyon (approximately 900.0 m in diameter) separates the caves. Terra Ronca I extends 700 m and has a cave entrance that is 96 meters high and 120 meters wide, with a religious altar measuring 76 meters long and 100 meters high where the religious ceremony of “Bom Jesus da Lapa” occurs every year in August. Terra Ronca II extends approximately 5.1 km, has an entrance of approximately 70 meters. Terra Ronca I and Terra Ronca II caves harbor several endemic animals and troglomorphic species.

### 2.2 Sampling

Samplings were conducted in April and October—2016 (license no 28992‐11 (ICMBio/SISBIO) and no 14886/2010 (Secima, Goiás)). At least two samples (replicates), composed of squares of approximately 0.25 m^2^, were collected in three areas of the caves: surface environment (outside of the cave), entrance cave (transition zone), and subterranean area (dark zones). Sampling zones were classified according to Culver and Pipan (2019). Approximately 300 g of the substrate (soil or cave sediment) was collected at five different points in each square to form a composite sample. The substrate was collected from a depth of 0 to 10 cm with the aid of a gardening shovel and stored in sterile plastic bags. The samples were transported to the laboratory in coolers, homogenized, sieved (2 mm mesh), and stored in a refrigerator at 4°C.

### 2.3 Physical and chemical parameters

Temperature (°C), air humidity (%), and luminosity (Lux) were measured at each sampled area with a minimum time interval of 1 min between measurements (Thermo‐hygrometer Instruntherm THAL‐300, 0.1 resolution, and ±5.0% accuracy). Rainfall data over 30 days before sampling were obtained from the Meteorological Database for Teaching and Research of the National Institute of Meteorology (Posse‐GO meteorological station, approximately 90 km from PETeR). Substrate pH was measured at a substrate: water ratio of 1:2.5 (w/w). The moisture in the soil and cave sediment samples was estimated by the gravimetric method, which involved drying at 105°C for 20 hr to 7 days after sampling, and the results were expressed as the dry weight percentage. Organic carbon (OC) concentration data were measured colorimetrically as described by Bartlett and Ross (1988). Total nitrogen (TN) was determined by Kjeldahl digestion followed by ammonia distillation (indophenol blue method) (Bremmer & Mulvaney, 1982). The composition of the subterranean substrate was carried out by scanning electron microscopy (*SEM*), together with chemical analysis by energy dispersive spectroscopy (EDS). An Oxford EDS coupled to a FEI Quanta 250 *SEM* was used to examine the chemical composition of the samples. The substrate was adhered to a double‐sided copper tape mounted on an aluminum stub to be observed (Zhai et al., 2012). About 15 ESEM images and corresponding EDS spectra of elements were acquired for each sample.

### 2.4 Microbiological parameters

Microbial biomass extraction was performed with 0.5 M K_2_SO_4_ in 10.0 g substrate samples after cell lysis by irradiation (Islam & Weil, 1998). Microbial biomass carbon (MBC) was evaluated by the irradiation extraction method (Brookes, Powlson, & Jendinson, 1982; Islam & Weil, 1998; Moura, Garrido, Sousa, Menezes, & Sampaio, 2018). The MBC contents were estimated with a spectrophotometer using the correction factor (K_CE_) of 0.41, as recommended for tropical soils, to avoid overestimating the results (Babujia, Hungria, Franchini, & Brookes, 2010). Microbial biomass nitrogen (MBN) was evaluated as described by Brookes, Landman, Pruden, and Jenkinson (1985). The MBN was determined by the addition of 1.5 ml of H_2_SO_4_ and 50 mg of a catalyst mixture (K_2_SO_4_ + CuSO_4_, 10:1) to 20 ml of substrate extract. The MBN concentrations were determined by spectrophotometry using the K_NE_ correction factor of 0.54 (Brookes et al., 1985).

Microbial respiration was determined by the quantification of the CO_2_ released by samples incubated in Bartha respirometric flasks (Bartha & Pramer, 1965). Samples (50.0 g substrate previously adjusted to 40% moisture content) were incubated in triplicate, and the CO_2_ was trapped in 30.0 ml of 0.5 N NaOH. For each analysis, there were two control flasks without substrate and only NaOH solution. The flasks were sealed and incubated in the dark at 25 ± 2°C for 48 hr. After this, microbial respiration was determined by the addition of saturated BaCl_2_ to the NaOH solution, followed by titration of the nonconsumed NaOH with 0.2 N HCl.

### 2.5 Statistical analysis

Data were analyzed by basic descriptive statistics (Shapiro–Wilks). Analysis of variance and Student's *t* tests with a 5% probability threshold were also applied to verify the significance of the differences among the results. To analyze the relationship between variables, Pearson's correlation coefficient was used, considering variables positively correlated with r ≥0.90. RStudio (2018) was used for the statistical analyses and production of graphs.

## 3 RESULTS

The total precipitation in April 2016 and October 2016 was 129.80 mm and 19.00 mm, respectively. There was no significant difference in the temperature and air humidity of the subterranean environments between the seasons. In general, subterranean environments had higher air humidity than surface areas. Substrate moisture was higher in the wet season than in the dry season at all sampling sites (Table 1). Surface areas showed higher substrate neutrality (pH close to 7.0). In contrast, the pH values of the entrance area and subterranean environments exhibited a greater degree of variation (6.93 to 9.02).

**TABLE 1:** Mean values and standard deviations of physical parameters (substrate moisture, air temperature, air humidity, and luminosity) in Angélica (A), São Bernardo (S), Terra Ronca I (T), and Terra Ronca II (TR) caves on the surface, entrance, and subterranean sample sites during wet (April 2016) and dry (October 2016) seasons

Cave		Substrate moisture (%)		Temperature (°C)		Air humidity (%)		Luminosity (Lux)	
		Wet	Dry	Wet	Wet	Wet	Dry	Wet	Dry
Angélica	Surface	11.07 ± 4.26	13.16 ± 2.25	26.78 ± 0.38	25.58 ± 0.46*	87.63 ± 3.49	77.70 ± 1.52*	458.33 ± 48.6	815.0 ± 76.6
	Entrance	1.04 ± 0.36	0.86 ± 0.37	30.46 ± 1.75	25.60 ± 0.73*	69.68 ± 3.94	76.51 ± 1.80*	34.16 ± 14.1	341.1 ± 51.6
	Subterranean	16.04 ± 1.64	13.23 ± 6.57	28.21 ± 0.24	27.75 ± 1.73	89.21 ± 5.86	83.88 ± 3.24	—	—
São Bernardo	Surface	3.55 ± 0.17	1.88 ± 0.58*	26.03 ± 1.06	28.63 ± 0.26*	97.55 ± 1.77	56.23 ± 2.63*	724.83 ± 113.6	514.3 ± 163.4
	Entrance	4.37 ± 1.58	8.55 ± 6.76	27.76 ± 0.76	27.51 ± 0.67	90.78 ± 2.77	76.33 ± 3.21*	151.83 ± 67.9	5.7 ± 2.2
	Subterranean	10.99 ± 6.95	1.40 ± 0.82*	26.03 ± 0.76	25.50 ± 0.91	96.63 ± 3.11	89.55 ± 3.99	—	—
Terra Ronca I	Surface	5.60 ± 3.85	1.13 ± 0.56*	26.85 ± 0.53	29.25 ± 0.10*	73.01 ± 4.24	68.86 ± 0.95*	1835.33 ± 71.8	879.6 ± 161,6
	Entrance	4.03 ± 2.54	5.86 ± 1.91	28.33 ± 0.55	30.96 ± 2.30*	66.23 ± 0.97	64.81 ± 3.93	43.16 ± 9.3	233.8 57.3
	Subterranean	3.39 ± 1.99	5.91 ± 1.32*	26.63 ± 0.98	27.38 ± 0.21	76.71 ± 13.96	68.11 ± 1.66	—	—
Terra Ronca II	Surface	8.21 ± 1.04	4.18 ± 0.94*	29.51 ± 0.42	26.96 ± 1.28*	70.83 ± 1.76	81.06 ± 12.23	209.6 ± 6.3	422,1 ± 72.2
	Entrance	0.73 ± 0.16	0.62 ± 0.60	27.75 ± 1.55	26.70 ± 0.30	75.53 ± 5.01	78.40 ± 5.70	13.5 ± 4.4	55.5 ± 24.4
	Subterranean	15.49 ± 4.51	12.56 ± 1.54	25.78 ± 0.27	25.78 ± 0.24	87.00 ± 1.69	89.71 ± 1.03	—	—

Note

Values with asterisks in a row differ between seasons with significance *p* < .05.

All caves had more organic carbon (OC) on the surface than in the other sampled areas, except the “T” cave, which had high OC values in the entrance area. Also, all caves (except the “T” cave) had significant differences in the amount of OC between wet and dry seasons. There was no pattern in the variation of the nitrogen amount in the sampled sites. Subterranean environments (“S”, “T”, and “TR” caves) showed a lower amount of nitrogen (N) in the dry season than in the wet season. Instead, the subterranean area of the “A” cave had a greater amount of N in the dry season. However, there was a significant difference in N concentration in all the subterranean areas between the wet and dry seasons (Table 2). The main chemical composition of the substrate was silica (Si), but it was not observed large concentrations of phosphorus and sulfur in subterranean samples (Table 3).

**TABLE 2:** Mean values and standard deviations of abiotic parameters (pH, nitrogen (N), and organic carbon (OC)) in Angélica (A), São Bernardo (S), Terra Ronca I (T), and Terra Ronca II (TR) caves on the surface, entrance, and subterranean sample sites during wet (April 2016) and dry (October 2016) seasons

Cave		pH		N (mg/kg)		OC (mg/kg)	
		wet	dry	wet	dry	wet	dry
Angélica	Surface	7.67 ± 0.22	8.22 ± 0.04 *	0.067 ± 0.039	0.036 ± 0.001	749.59 ± 517.79	473.11 ± 36.00
	Entrance	6.93 ± 1.03	8.33 ± 0.31 *	0.015 ± 0.0028	0.195 ± 0.175*	163.04 ± 58.70	154.03 ± 53.74
	Subterranean	8.16 ± 0.46	8.71 ± 0.28 *	0.016 ± 0.0022	0.024 ± 0.0013**	113.20 ± 8.64	79.01 ± 15.11**
São Bernardo	Surface	7.61 ± 0.11	7.40 ± 0.42	0.113 ± 0.080	0.039 ± 0.007*	346.30 ± 52.61	292.78 ± 42.53
	Entrance	7.78 ± 0.66	8.53 ± 0.14 *	0.032 ± 0.022	0.024 ± 0.018	201.14 ± 131.54	269.66 ± 203.36
	Subterranean	8.32 ± 1.09	8.61 ± 0.61	0.020 ± 0.002	0.015 ± 0.00**	77.04 ± 11.36	44.22 ± 14.18**
Terra Ronca I	Surface	8.88 ± 0.14	8.69 ± 0.29	0.055 ± 0.040	0.075 ± 0.047	820.37 ± 71.61	824.48 ± 700.84
	Entrance	7.49 ± 1.58	7.98 ± 0.75	0.078 ± 0.059	0.025 ± 0.002*	1,008.20 ± 700.86	1,170.71 ± 1,044.51
	Subterranean	7.69 ± 0.25	7.59 ± 0.10	0.036 ± 0.004	0.025 ± 0.007**	448.93 ± 145.67	334.98 ± 51.23
Terra Ronca II	Surface	7.85 ± 0.44	7.63 ± 0.27	0.025 ± 0.016	0.049 ± 0.041	431.34 ± 367.63	708.91 ± 134.31
	Entrance	8.37 ± 0.82	8.51 ± 0.62	0.008 ± 0.002	0.025 ± 0.007**	85.07 ± 6.60	197.55 ± 10.88**
	Subterranean	9.02 ± 0.40	7.56 ± 1.81	0.052 ± 0.009	0.032 ± 0.001**	176.91 ± 13.28	299.38 ± 17.52**

Note

Values with asterisks in a row differ between seasons with significance *p* < .05.

**TABLE 3:** Mean values and standard deviations of the chemical composition of the substrate (wt ‐ %) in Angélica (A), São Bernardo (S), Terra Ronca I (T), and Terra Ronca II (TR) caves on the subterranean sites during wet (April 2016) and dry (October 2016) season

Caves	Season	O	Si	Al	Fe	Cu	K	Mg	Ca	S	P
Angélica cave	wet	58.9 ± 3.1	28.4 ± 5.6	3.2 ± 1.6	1.7 ± 0.8	0.8 ± 0.4	0.6 ± 0.2	0.3 ± 0.2	0.1 ± 0.1	—	—
	dry	51.9 ± 2.2	18.1 ± 3.5	1.6 ± 0.2	0.7 ± 0.2	0.7 ± 0.3	0.2 ± 0.09	0.2 ± 0.05	0.07 ± 0.05	—	—
São Bernardo cave	wet	58.1 ± 5.2	30.0 ± 9.7	2.1 ± 1.1	1.1 ± 0.6	0.8 ± 0.1	0.2 ± 0.2	0.1 ± 0.1	0.1 ± 0.1	—	—
	dry	60.5 ± 0.7	35.0 ± 1.2	1.6 ± 0.4	0.9 ± 0.5	1.2 ± 0.6	0.07 ± 0.1	—	—	—	—
Terra Ronca I cave	wet	57.3 ± 4.6	24.2 ± 7.7	3.5 ± 1.5	1.4 ± 0.4	1.3 ± 1.0	0.7 ± 0.4	0.3 ± 0.1	0.2 ± 0.1	—	—
	dry	58.5 ± 4.8	26.9 ± 8.9	2.4 ± 0.3	0.9 ± 0.01	0.7 ± 0.6	0.5 ± 0.3	—	0.1 ± 0.05	—	—
Terra Ronca II cave	wet	58.9 ± 4.4	10.1 ± 8.8	3.6 ± 1.1	1.7 ± 1.1	1.45 ± 0.4	0.5 ± 0.4	11.8 ± 2.5	3.1 ± 2.4	0.08 ± 0.02	0.01 ± 0.04
	dry	59.2 ± 3.5	16.6 ± 3.5	2.7 ± 1.8	1.3 ± 1.0	1.6 ± 0.8	0.3 ± 0.2	8.3 ± 0.8	2.6 ± 1.1	0.07 ± 0.01	0.02 ± 0.01

**Figure 1:**
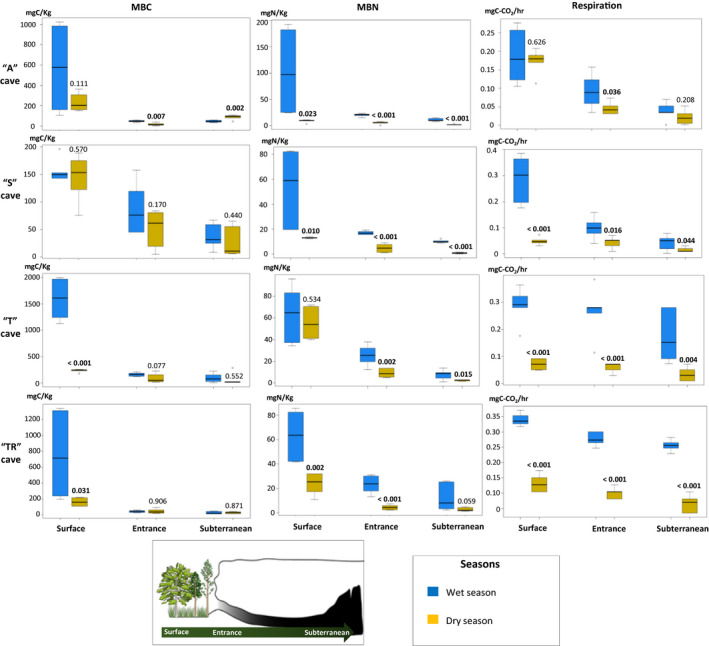
Mean values of microbial biomass carbon (MBC), microbial biomass nitrogen (MBN), and respiration rate in soil and cave sediment in Angélica (“A” cave), São Bernardo (“S” cave), Terra Ronca I (“T” cave), and Terra Ronca II (“TR” cave) caves on the Surface, Entrance and Subterranean sites (see schematic illustration) during wet (April 2016, blue boxplots) and dry (October 2016, yellow boxplots) seasons. The values above the yellow box represent p‐values for the difference between dry and wet seasons (*p* < .05 in bold)

The mean MBC values were higher on the surface than in the entrance and subterranean areas of the all caves in both seasons (Figure 1), but there was no difference in the amount of MBC in subterranean sites between the seasons, except in “A” cave where the MBC was higher in the dry season than in wet season. There was a decrease in the amount of microbial biomass nitrogen (MBN) between the surface, entrance, and subterranean areas of the same cave (Figure 1). The MBN values on the surface of the "A" and "S" caves varied more substantially than those on the other sites. The MBN values were lower in the dry than in the wet season when data showing significant differences between the seasons were evaluated.

Substrate microbial activity was measured by the microbial respiration rate (Figure 1). Respiration rates were significantly higher in the wet season. The "T" and "TR" caves stood out with the highest rates of respiration in all the areas sampled. However, there was no significant difference in microbial respiration values between the sampled areas of these caves during the dry season. The surface of the "A" cave showed higher values of microbial respiration than those found in the entrance and subterranean areas. In contrast, the "S" cave presented similar respiration rates between the sampled sites, highlighting only the high value of microbial respiration on the surface of the "S" cave in the wet season.

**Figure 2:**
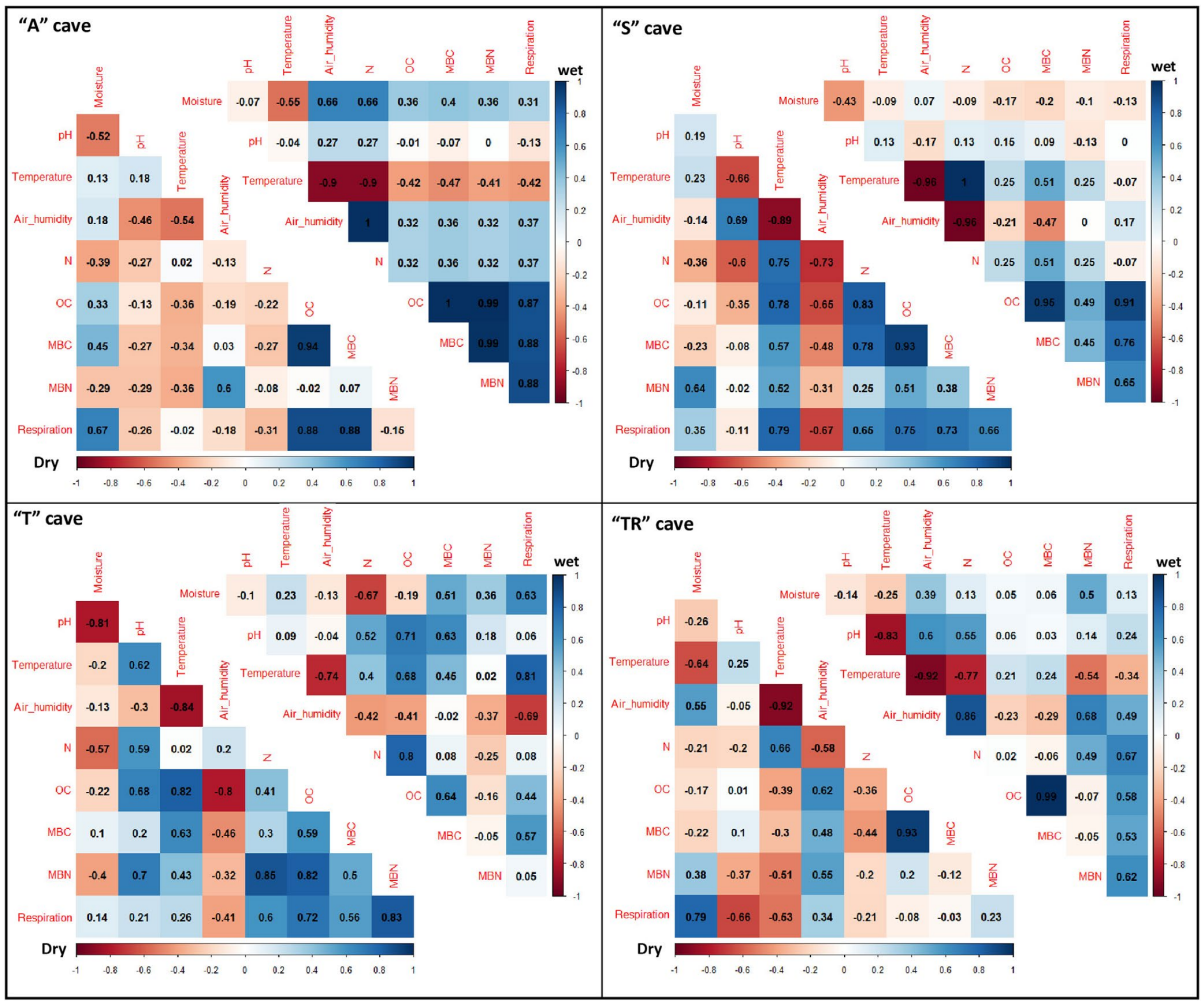
Correlogram with Pearson correlation coefficient values between physical, chemical, and microbiological parameters (substrate moisture, pH, temperature, air humidity, nitrogen (N), organic carbon (OC), microbial biomass carbon (MBC), microbial biomass nitrogen (MBN), and respiration) from subterranean environments in Angélica (A), São Bernardo (S), Terra Ronca I (T), and Terra Ronca II (TR) caves during wet (April 2016) and dry (October 2016) seasons

All caves, except the "A" and “TR” cave, showed a strong positive correlation between MBC and OC in both seasons (Figure 2). The subterranean habitat of the “A” cave presented a significant negative correlation between microbial biomass nitrogen (MBN) and MBC, even with a positive correlation between OC and MBN, during both seasons.

## 4 DISCUSSION

The literature reports a wide pH range, from near neutral to slightly alkaline, in the karst (Zhang, Li, Pan, & Ren, 2006) that was also observed in this study. Highly alkaline areas (such as the “SB” cave) show the influence of water on the dissolution of the carbonate rock, raising substrate pH values. Acidic substrates inside the cave (such as the “A” cave) may originate from the different intermixing ratios of the weathering residue of carbonate rock, sandy rock, or shale components and decomposed organic matter (Tian et al., 2008; Yang et al., 2010).

Higher values of organic carbon and total nitrogen were observed in surface areas because this location has high accumulation and richness of organic matter sources. Many caves in temperate regions occupy the lower values of the carbon input range seen globally, and their resident communities are generally energy‐starved (<5.0 mgC/L) (Chelius et al., 2009; Northup & Lavoie, 2001; Ortiz et al., 2014). However, all the caves in this study showed more organic carbon than those found in temperate caves. High organic carbon values in tropical caves have also been observed in previous studies (Paula et al., 2016). Although this difference occurs, temperate and tropical cave communities are supported by organic inputs that originate from outside or allochthonous sources. Simon et al. (2007) created a carbon flow model in caves demonstrating that the input of organic carbon into the caves occurs mainly by small openings, cracks, and sinks that allow the entry of leaves, wood, and debris from streams and epikarstic environments. The caves studied in this work have large rivers that run through the surface before entering the cave. Thus, according to the model of Simon et al. (2007), these rivers carry surface organic matter into the cave (particulate and dissolved organic carbon) that accumulates in the sediment bank, mainly in dry seasons. The “A” and “S” caves are a good example where the highest precipitation in the wet season leaches out a larger amount of organic carbon to the substrate, whereas in the dry season, there is greater heterogeneity in the amount of carbon along with the subterranean environment. The same pattern was observed in the Kartchner cave, with higher organic carbon during the rainy season (Ortiz et al., 2014).

Although the caves studied presented nitrogen, no significant amounts of sulfur and phosphorus were found in the subterranean samples. Several nutrients, such as nitrogen, sulfur, and phosphorous, are considered limiting factors in subterranean environments (Goldscheider, Hunkeler, & Rossi, 2006). Areas farther from the entrance zone of these caves showed smaller variations in the nitrogen amount, as observed in Lapa Nova cave (Pellegrini & Ferreira, 2013). The presence of high amounts of nutrients in the caves (nitrogen, phosphorous, and sulfur), from endogenous (autochthonous) or exogenous (allochthonous) sources, can have a profound impact on the fauna, microbial growth, and community structure (Jonhston, Muench, Banks, & Barton, 2012). Sulfur and phosphorus may be present, originating from the mineral matrix of the rock, depending on the cave geochemistry, but any intrinsic source of nitrogen in caves is rare (Klimchouk, 2000). In our work, the amount of nitrogen was higher in the wet season than in the dry season, similar to the levels observed in the organic carbon. This can be explained by the greater intensity of surface soil leaching and by flash floods into the cave in this period. The subterranean area of the "A" cave was the only one that presented higher nitrogen values in the dry season in contrast to the wet ones. The main nitrogen sources in subterranean environments are plant residues, crop stubble, natural vegetation (carried by rainwater or streams surface), or animal excretion (Dubey, Kaur, Suryanarayana, & Murthy, 2014).

The surface area also had higher microbial biomass values than those inside the cave. All the caves showed microbial biomass values similar to previous studies in subterranean environments worldwide (Carmichael et al., 2015; Paula et al., 2016). There was a strong positive correlation between microbial biomass and organic matter, indicating a great influence of the quantity (amount of C) and quality (amount of N) of organic matter in microbial community biomass. Previous studies have demonstrated that microbial biomass within caves is generally low compared with surface habitats (Barton & Jurado, 2007). The putative reasons for this include the low rate of inputs and low diversity of the autochthonous and allochthonous organic substrates (Chelius et al., 2009).

Recent studies suggest the use of soil microbial biomass as an ecosystem limitation indicator (Schloter et al., 2018; Xu, Thornton, & Post, 2013) and affirm that this parameter reflects the degree of immobilization of carbon and nitrogen. The relationship between MBC and organic carbon or MBN and nitrogen can be interpreted as substrate available and the portion of carbon and nitrogen immobilized in microbial cells. Therefore, the dynamics of microbial biomass can be a useful parameter to monitor the availability of organic matter that is less recalcitrant (Anderson & Domish, 1993). A decrease in microbial biomass could result in the mineralization of nutrients, while an increase in microbial biomass may indicate the immobilization of nutrients (McGill, Gannon, Robertson, & Cook, 1986).

Microbial biomass carbon (MBC) decreased in the dry season on the surface, while there was no reduction in the subterranean environment. Microbial biomass nitrogen (MBN), on the other hand, decreased in the dry season, both in the surface (except “T” cave) and subterranean environments. A lack of changes in MBC indicates that there were no alterations in the immobilization of the carbon to microbial cells and consequently in carbon mineralization. Although MBC did not show variations with the season, the amount of MBN was higher in the wet season, indicating higher nitrogen mineralization in the dry season and greater biomass accumulation in the wet season. Considering the reduction of the amount of organic carbon in the dry season and the dynamics of MBC, there was a higher rate of carbon mineralization of the microbial biomass in surface communities. Subterranean microbiota in the "S", "T", and "TR” caves did not show significant differences in the amount of microbial biomass carbon, even with the organic carbon reduction. On the other hand, the microbial community within the "A" cave increased the microbial biomass carbon, even with a reduction in the amount of organic carbon in the dry season. Furthermore, the amount of microbial biomass nitrogen decreased according to the availability of nitrogen, except in the "A" cave, which reduced MBN even with more nitrogen in the dry season. Generally, higher availability of nutrients in the environment leads to greater mobilization into local microbial biomass and thus contributes to nutrient pools. The high availability of nitrogen fractions may explain the high microbial biomass in the depositional site, such as the “S”, “T”, and “TR” caves in the wet season. However, large input and fast turnover of relatively fresh and labile nitrogen could stimulate the mineralization of old organic matter as well, which might be a direct explanation for the low concentrations of MBN in the depositional site (Li et al., 2015), such as the “A” cave.

Microbial respiration represents CO_2_ release by microbial organic matter and litter decomposition (Schindlbacher, Zechmeister‐Boltenstern, & Jandl, 2009; Zhou et al., 2014). Higher microbial respiration was found on the surface area of the caves. Microbial respiration inside the caves varied positively according to organic carbon availability and microbial biomass variation. In general, the amount of organic matter (carbon and nitrogen) and microbial biomass was higher in the wet season, with an increase in microbial respiration. All these factors agree with a greater immobilization of organic matter into microbial biomass and a reduction of the microbial growth in the dry season, as described above. The “A” cave showed higher microbial respiration values with higher amounts of organic carbon and MBN together with lower values of nitrogen in the wet season. When growing on N‐poor substrates, microorganisms do not have enough nitrogen to build up as much biomass as the carbon concentration would allow. Thus, it has been argued that microorganisms can dispose of carbon via overflow respiration as CO_2_ so that the substrate meets their nutritional demands (Sinsabaugh et al*.*, 2013). Overflow respiration in ecosystems, that is, respiration without the production of energy, has recently been questioned by several studies. First, it has been found that it may be more beneficial for the microorganisms to eliminate excess carbon by releasing as dissolved organic carbon than to expend energy in the respiratory chain (Spohn, 2015). Second, it has been pointed out that the energy lost by the disposition of carbon could be invested in storage or other processes, which increase the fitness of the microorganism (Hessen, Elser, Sterner, & Urabe, 2013).

The dynamics of the microbial biomass in the subterranean areas of the "A", "S", "T", and "TR" caves could be framed in two theories—“stoichiometric decomposition” and “microbial nitrogen mining”—regarding the impacts of nutrient availability on organic matter decomposition (Craine, Morrow, & Fierer, 2007). The "S", "T", and "TR" caves could fit the “stoichiometric decomposition” theory, where the microbial activity is highest, and the decomposition rates are maximal if the carbon and nitrogen inputs with substrate match the microbial demands. The “A” cave would fit into the “microbial nitrogen mining” theory, which assumes that the N‐acquiring microbes use labile carbon as an energy source to decompose recalcitrant organic matter, which contains nitrogen (Craine et al., 2007; Moorhead & Sinsabaugh, 2006). This means that low‐N pool availability for microorganisms facilitates the decomposition of recalcitrant organic matter to acquire nitrogen. In short, according to “stoichiometric decomposition,” high‐N availability (nutrient‐rich condition) is likely to be beneficial for organic matter decomposition, while according to “microbial N mining,” low‐N availability (nutrient‐poor condition) is likely to facilitate recalcitrant organic matter decomposition (Chen et al., 2014). However, further studies on the stock and flow of carbon and nitrogen in these caves are needed to improve the understanding of the metabolism of these compounds.

## 5 CONCLUSIONS

Although physical and chemical parameters are widely used as terrestrial and subterranean environmental indicators, microbiological parameters in caves, such as respiration and microbial biomass, can design the environmental quality scenario and respond more quickly to changes in caves. The microbial parameters in this study were good indicators for evaluating the nutrient dynamics in the studied environments, and with this knowledge, we can perform better health monitoring to follow the changes in these habitats.

Differences in the availability of C and N found between the “A”, “S”, “T”, and “TR” caves may have shaped the microbial community strategies for organic matter decomposition and the incorporation of these elements into their biomass. Therefore, the caves studied showed different dynamics of microbial biomass, which allows them to be considered unique and possess specific characteristics. Knowledge of the subterranean environments and local ecosystem processes is still very incipient, with few studies on nutrient flux in subterranean environments. Considering the specificity of each subterranean habitat, the study of each cave is extremely important to understand the local community (microorganisms and fauna) and the dynamics of energy flow in food webs. Thus, research developed in this scope would contribute to better use and management of this environment, allowing us to indicate areas more fragile to economic and/or ecotourism exploration and help adopt safer decisions about the conservation of these sites.

## CONFLICT OF INTERESTS

None declared.

## AUTHOR CONTRIBUTIONS

Caio Paula involved in conceptualization, formal analysis, funding acquisition, investigation, methodology, and writing‐original draft; Maria Bichuette involved in conceptualization, investigation, writing‐review, and editing; Mirna Seleghim involved to conceptualization, funding acquisition, investigation, writing‐review, and editing.

## ETHICS STATEMENT

None required.

## Data Availability

All data generated or analyzed during this study are included in this published article. On request, additional data can be obtained from the corresponding author.
